# Hints for Using Iodoform

**Published:** 1881-12

**Authors:** B. M. Walker

**Affiliations:** Danville, Va.


### HINTS FOR USING IODOFORM.

*Ed. Med. and Surg. Reporter*:—

It has been a great desideratum with the profession to find a deodorizer for iodoform. I have used in my practice a solution of hydrate of chloral ʒj to ℥j of water, as a wash for the hands when I wish to remove the odor, which it unquestionably does in a few moments. I have also used equal parts of the hydrat. chloral and iodoform, say ʒj each, glycerine ʒij, ung. petrolii ʒvj, as an ointment to promote granulation in unhealthy ulcers. This combination was highly satisfactory in the object desired; the ulcer healing kindly, and the disagreeable odor entirely destroyed. In cases requiring the powder of iodoform in a dry state, let the absorbent cotton be wet with this solution of chloral and dried, then placed upon the parts that are covered with the iodoform, and the odor will be very effectively controlled, indefinitely better than the combination of bals. Peruvian, mint, or anything else I have tried. In not a few instances the chloral will prove a factor in the treatment. B. M. WALKER, M. D.

*Danville, Va.*

